# Internal Density Calibration in the Proximal Humerus to Estimate Bone Stiffness for Stemless Shoulder Arthroplasty

**DOI:** 10.1002/jor.70143

**Published:** 2026-01-15

**Authors:** Chloë K.A. Stiles, Bryn E. Matheson, George S. Athwal, Jack P. Callaghan, Clark R. Dickerson, Steven K. Boyd, Nikolas K. Knowles

**Affiliations:** ^1^ Kinesiology and Health Sciences University of Waterloo Waterloo Ontario Canada; ^2^ McCaig Institute for Bone and Joint Health University of Calgary Calgary Alberta Canada; ^3^ Roth McFarlane Hand and Upper Limb Centre London Ontario Canada

## Abstract

End‐stage osteoarthritis (OA) alters bone density in the humeral head, complicating implant fixation during stemless shoulder arthroplasty. Current preoperative assessments fail to consider the mechanical properties of bone directly supporting the humeral component. While in‐scan phantom calibration is used to determine volumetric bone mineral density (vBMD), phantoms are rarely used clinically. An internal density calibration method has been developed, but not yet applied in the proximal humerus. This study examined correlations between phantom and internal density calibration, and between vBMD and estimated stiffness in the proximal humerus. Nonpathologic cadaveric CT images containing a K_2_HPO_4_ phantom were used to analyze a 10 mm region of interest below the anatomic neck. Phantom calibration was performed. Internal calibration used air (A), adipose (Ad), skeletal muscle (M), and cortical bone (C) in three combinations (AAdCM, ACM, AAdC). Finite element models (FEMs) were generated from each. Strong correlations were observed between phantom‐ and internally calibrated vBMD (AAdC *R*² = 0.80; AAdCM *R*² = 0.88; ACM *R*² = 0.90), with ACM showing the lowest error (9.98%). Estimated stiffness and vBMD were strongly correlated across calibrations (*R*² = 0.61–0.66), with ACM showing the lowest error (5.46%). Findings support internal calibration for determining vBMD and FEMs for estimating stiffness in the proximal humerus.

## 1 Introduction

Shoulder arthroplasty has become widely regarded as an effective surgical treatment for glenohumeral joint degeneration due to bone diseases such as osteoarthritis (OA) [1, 2]. In the shoulder, OA is characterized by degeneration of articular cartilage leading to morphological changes in the subchondral bone of the humerus and glenoid [3, 4]. With pathologic progression, these changes alter bone mineral density (BMD) within the humeral head [3].

BMD is strongly correlated with bone stiffness and strength in OA populations [5, 6, 7], and reductions in BMD compromise mechanical integrity. This loss in strength poses a risk for implant stability, increasing the likelihood of postoperative complications and the need for early surgical revision [6, 8, 9]. Despite its recognized importance, there is no standardized metric for determining bone mechanical properties, namely stiffness or strength, in end‐stage OA [10, 11]. While several studies have compared diseased bone relative to nondiseased bone in the humeral head [2, 12, 13, 14], validated models that link BMD and mechanical properties of bone in patients undergoing shoulder arthroplasty remain limited. No studies have evaluated the bone, directly below the resected humeral head, that provides support to humeral head components used in shoulder arthroplasty.

Stemless humeral head components, consisting of a metal hemisphere with no diaphyseal stem, achieve primary fixation by anchoring to the metaphyseal trabecular bone directly below the resected plane at the anatomic neck [15, 16, 17]. These components are increasingly used in shoulder arthroplasty for end‐stage OA as they aim to preserve nondiseased bone in the proximal humerus for future surgical revisions [11, 18]. However, current preoperative clinical imaging assessments are limited in their ability to quantitatively assess volumetric bone mineral density (vBMD) or to characterize the mechanical competence of bone directly supporting the component [10], instead relying on subjective intra‐operative evaluation of the resected bone surface. This leads to difficulty in pre‐operatively selecting which patients are good candidates for stemless components and which patients would benefit from the increased stability associated with a stemmed component.

Single‐energy computed tomography (CT) is commonly used for pre‐operative planning to visualize bone deformities and to template component size and positioning. These CT images provide voxel‐level estimates of tissue density, proportional to the attenuation of the X‐ray beam through the tissue, in Hounsfield units (HU) [19]. HUs can be variable due to differences in scanner manufacturers, acquisition settings, and reconstruction protocols [19, 20]. To address this variability, the CT scan's native HUs can be standardized by calibrating the scan HUs against a calibration phantom containing materials of known densities. This process, known as phantom calibration, converts HUs to bone equivalent density, enabling derivation of quantitative CT (QCT)‐based vBMD measurements [21, 22]. These CT images calibrated to vBMD measurements can be used as input to image‐based finite element models (FEMs) to estimate bone mechanical properties [22].

Phantom calibration uses a dipotassium phosphate (K_2_HPO_4_) or hydroxyapatite (HA) phantom containing rods of materials with known densities to calibrate single‐energy CT images. Calibration is used to establish a linear standardized relationship between voxel‐specific intensity (HU) to units of radiological density (vBMD) to provide equivalent density in units of mg_K2HPO4_/cm^3^ (K_2_HPO_4_ phantom) or mg_HA_/cm^3^ (HA phantom) [23]. However, phantom calibration is seldom used clinically due to practical limitations [24, 25].

Recently, an internal density calibration method has been developed that uses tissues with known densities as references to convert CT scans into bone equivalent density images [25]. While this method was developed to evaluate vBMD and stiffness in the hip and spine, it has never been tested in the proximal humerus. This evaluation is needed since attenuation values and related bone stiffness are skeletally site‐specific [26, 27] and are affected by bone quality and pathology [22]. Additionally, differences in shoulder anatomy, relative to hip and spine anatomy, may lead to challenges with the availability of different referent tissues for the internal calibration method. Once the relationship between vBMD from the internal calibration method and the phantom calibration method has been established, internal density calibration can be used to quantify vBMD.

The primary objective of this study was to evaluate the agreement between vBMD derived from phantom‐based calibration and vBMD obtained through internal calibration methods, using three different combinations of reference tissues in the proximal humerus. The secondary objective was to assess the relationship between vBMD and FEM–estimated stiffness. We hypothesized that internal calibration would produce vBMD values comparable to those from phantom calibration, and that vBMD would be strongly correlated with estimated stiffness.

## 2 Methods

### 2.1 Cadaveric Images

Single‐energy CT images from thirty‐nine nonpathologic cadaveric shoulder specimens were selected from our pre‐existing lab database based on the criteria of containing a liquid K_2_HPO_4_ calibration phantom (Model 3 CT Calibration Phantom, Mindways Software Inc., Austin, TX, USA) in the scan field of view. Each scan was performed on a GE Discovery CT750 HD scanner located at the Roth McFarlane Hand & Upper Limb Centre (London, Ontario) at 120 kVp, 200 mA, 0.625 mm slice thickness, 512 × 512 image resolution, and pixel spacing ranging from 0.547 to 0.902 mm. All images were reconstructed using the Boneplus reconstruction kernel, which provides high spatial resolution and contrast within the image [28]. This image protocol is consistent with pre‐operative CT imaging used at our center.

### 2.2 Isolating the Volume of Interest (VOI)

**Figure 1:**
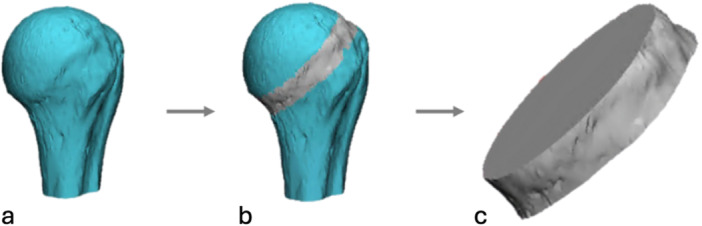
Method for isolating the 10 mm volume of interest. (a) A part was created of the proximal end of the humerus from the raw DICOMs (Mimics). (b) A 10 mm cuboid was aligned distal to the estimated resection plane (3‐Matic). (c) A Boolean intersection (Meshlab) was then applied to isolate the VOI for analysis.

The humerus was segmented in each image using a global threshold and standard HU window (HU > 225) (Mimics v.20.0, Materialise, Leuven, BE). Region growing and semi‐automated morphological tools were used to isolate and fill the proximal end of the humerus for vBMD measurements and FEM generation. A 10 mm region directly below the anatomic neck was isolated by aligning a 10 mm cuboid below the ideal plane of resection, simulating the bone directly supporting a stemless humeral component (3‐matic v.18.0, Materialise, Leuven, BE). A Boolean intersection was applied to isolate the common VOI for analysis across calibration methods (MeshLab v2023.12) (Figure 1).

### 2.3 Density Calibration Methods for Quantitative Analysis

#### 2.3.1 Phantom Calibration

**Figure 2:**
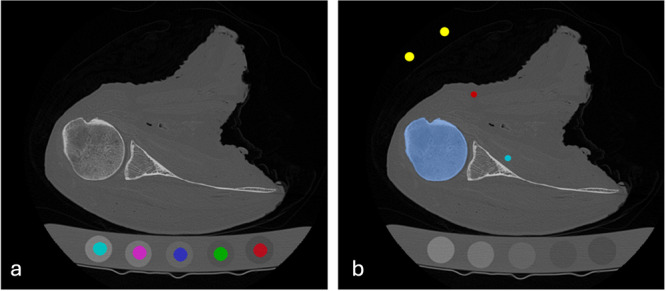
Density calibration methods for QCT image analysis. (a) Phantom calibration. K2HPO4 can be seen at the base of the image. Each rod is manually segmented 10 slices from each end and then interpolated (ITK‐SNAP) to establish the linear conversion between HU and K_2_HPO_4_ density using the highest (teal) and lowest (red) densities [25]. (b) Internal calibration. Referent tissues of air (yellow), adipose (red), and skeletal muscle (teal) were segmented manually at the level of the proximal humerus. Cortical bone (blue) was thresholded to determine the highest HU value (which is essential for establishing the HU‐density conversion).

Phantom calibration was performed by manually segmenting the phantom rods in each image according to standard procedure outlined in the Midways Model 3 QCT Phantom User Manual (ITK‐SNAP v4.2.0) [29]. After correcting for water as described in the user manual, a linear relationship was established between the in‐scan phantom HUs and the known K_2_HPO_4_ density (Figure 2a) [30]. Custom code (https://github.com/Bonelab/Ogo/) was used to produce calibrated QCT images in mgK_2_HPO_4_/cm^3^ [29].

#### 2.3.2 Internal Calibration

For the internal calibration, regions of interest (ROI) were manually selected for air (A), adipose (Ad), and skeletal muscle (M) as follows: air was taken outside the body, adipose was taken subcutaneous, and skeletal muscle was taken from the subscapularis, infraspinatus, or deltoid depending on the need to avoid muscle fat infiltration (Figure 2b). Blood was excluded as cadaveric tissues do not contain a defined blood‐filled artery for measurement, and there is not a large enough ROI within the field of view of the shoulder in patient images. ROI placement was at the same level as the proximal humerus VOI to reduce the potential for HU variations due to beam hardening [24, 30, 31]. The proximal humerus was segmented within each image using a semi‐automated method of applying a Hessian‐based bone enhancement filter combined with a graph cut technique [32, 33]. The bone was thresholded to determine the highest HU value between 1000 and 1500 HU for cortical bone (C) [34]. The mean HU value from each tissue ROI was extracted to determine whether they match expected HU values for each tissue (Table 1). Internal calibration was performed using three different referent tissue combinations (AAdC, AAdCM, ACM) to determine the combination with the lowest mean difference compared to phantom calibration [24]. The same source code used for the phantom calibration was used to produce internally calibrated QCT images in mgK_2_HPO_4_/cm^3^.

**Table 1:** Mean and standard deviation of HU values across specimens for each tissue ROI.

	Air (A)	Adipose (Ad)	Cortical bone (C)	Skeletal muscle (M)
Mean (HU)	−997.75	−55.29	1256.98	10.98
SD (HU)	1.61	22.33	14.40	29.98
Expected (HU)	−1000	−60	1200	40

*Note:* Values extracted from the QCT txt file. Expected HU values are based on values outlined in Chapter 2 of Radiology Secrets Plus, Third Edition.

The subject‐specific VOI was overlayed with both the phantom and internal density calibrated QCT images, and vBMD values were extracted (3D‐Slicer v.5.6.2) [35]. Results were compared for each internal calibration tissue combination to the phantom calibration using linear regression and Bland–Altman analysis.

### 2.4 FEM Creation

Using embedded tools (3‐matic v.18.0, Materialise, Leuven, BE), the isolated VOI was converted to a triangular surface mesh with an absolute maximum 1.5 mm edge length and 0.15 mm geometrical error, selected based on prior convergence‐validated meshing protocols specific to humeral bone found in the literature [36, 37, 38]. VOIs were then volume meshed with 4‐node linear tetrahedral elements (C3D4) using finite element software (Abaqus v.2022, Simulia, Providence, RI, USA). Material properties were assigned to the volume mesh by overlaying the subject‐specific and calibration method‐specific, density‐calibrated QCT image (Bonemat v.3.2) [39]. A K_2_HPO_4_‐based density modulus relationship, specific to human long bones ($E_{\text{bone}}=12,486\rho_{\text{qct}}^{1.16}$) [40, 41] was applied through the entire density range of the model. This equation was chosen for consistency with models created from the same anatomic location and is K_2_HPO_4_ specific [41], reducing bias in density conversion.

**Figure 3:**
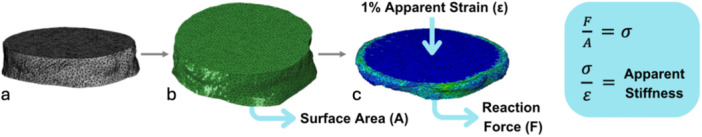
Finite element model generation. (a) Triangular surface mesh of VOI (3‐Matic). (b) Tetrahedral volume mesh with assigned material properties (Bonemat). (c) FEM with a 1% applied compression strain (Abaqus), magnified 10x for visualization.

FEMs were generated with the proximal and distal surfaces of the VOI acting as rigid bodies, with guiding nodes fixed to all nodes on the proximal and distal surfaces (Abaqus). The local coordinate system was aligned with the *x–y* plane along the proximal surface and the *z*‐axis pointing distally. A 1% apparent strain (0.1 mm displacement) [42] was applied along the *z*‐axis from the proximal surface with the distal surface fully fixed. The reaction force on the distal surface at the maximum applied displacement was collected Figure 3.

Apparent stress was calculated by taking the reaction force of the FEM simulation (F), divided by apparent area averaged across the proximal and distal VOI surfaces (A). Apparent stiffness was calculated as the apparent stress divided by the applied apparent strain. Four different FEMs were created for the thirty‐nine‐phantom containing cadaveric images for each different calibration method (AAdC, AAdCM, ACM, ROD).

### 2.5 Statistical Analysis

To evaluate the agreement between phantom and internal calibration methods, linear regression and Bland–Altman analyses were performed comparing vBMD values derived from each internal calibration tissue combination (AAdCM, ACM, AAdC) against the phantom calibration reference. The internal calibration tissue combination with the lowest mean difference and narrowest limits of agreement in the Bland–Altman plots was considered the best combination.

To examine the relationship between bone density and apparent stiffness, linear regression was used to assess the correlation between vBMD and stiffness across all subjects, separately for each calibration method. The agreement between stiffness estimates derived from phantom versus internal calibration was further assessed using Bland–Altman analysis.

For each linear regression analysis, model assumptions were evaluated as follows: linearity and homoscedasticity were assessed with residual‐versus‐fitted plots; normality of residuals with Q–Q plots and the Shapiro–Wilk test; and influential outliers with Cook's distance. Only mean bias and limits of agreement were reported for Bland–Altman analyses. Heteroscedasticity was assessed by regressing the absolute differences against the means in Bland–Altman plots. All statistics were performed using R studio (v2024.04.1 + 748).

## 3 Results

### 3.1 Regression Model Assumption Checks

Regression models generally met the linearity and homoscedasticity criteria. Residuals were normal for ACM (*p* = 0.15) and stiffness vBMD models (*p* = 0.68–0.85). Deviations from normality were observed in AAC (*p* = 0.0039) and AACM (*p* = 0.013) models, but Cook's distances were low (0.22–0.39), indicating no influential outliers. Standard linear regression results are reported.

### 3.2 Internal Calibration Tissue Combinations

The mean HU values for air, cortical bone, and adipose were all within the expected ranges (Table 1) for internal calibration, with air having the least amount of variability (SD = 1.61 HU) and skeletal muscle having the highest variability (SD = 29.98 HU).

**Figure 4:**
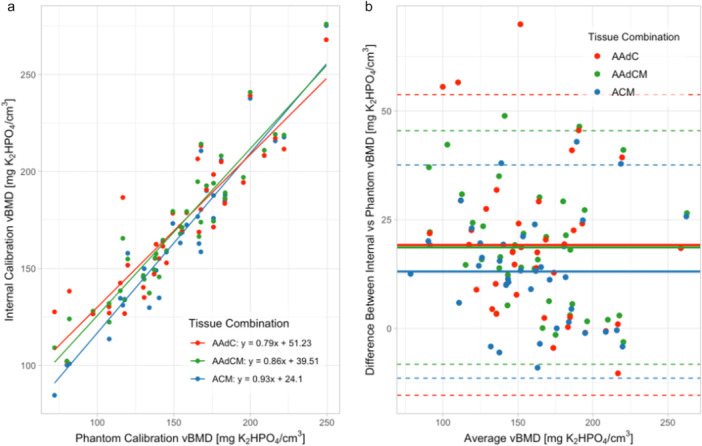
Comparing calibration techniques. (a) Linear regression showing correlation between vBMD (mgK_2_HPO_4_/cm^3^) from internal calibration according to tissue combination relative to phantom calibration (*n* = 39). (b) Bland–Altman plot for vBMD (mgK_2_HPO_4_/cm^3^) for internal calibration according to tissue combination relative to phantom calibration (*n* = 39).

Linear regression showed a strong correlation between vBMD values from phantom and internal density calibration (AAdC *R* ^2^ = 0.80; AAdCM *R* ^2^ = 0.88; ACM *R* ^2^ = 0.90) and a slope not significantly different from 1 (AAdC: *y* = 0.788x + 51.2; AAdCM: *y* = 0.862x + 39.5; ACM: *y* = 0.927x + 24.1) (*p* < 0.001) (Figure 4a). Bland–Altman analysis revealed the ACM tissue combination had the lowest error, with a mean bias of 13.1 mgK_2_HPO_4_/cm^3^ (9.98%) with 95% limits of agreement ranging from −11.4 to 37.6 mgK_2_HPO_4_/cm^3^ (Figure 4b, Table 2). On average, internal density calibration overestimated phantom‐based vBMD for all tissue combinations.

**Table 2:** Bland Altman results for each internal calibration tissue combination, relative to phantom calibration for vBMD.

	AAdC	AAdCM	ACM
Mean difference (mgK_2_HPO_4_/cm^3^)	19.18	18.61	13.08
Upper limit (mgK_2_HPO_4_/cm^3^)	53.74	45.46	37.60
Lower limit (mgK_2_HPO_4_/cm^3^)	−15.40	−8.23	−11.44
Percent error (%)	15.71	14.61	9.98

*Note:* The table shows mean differences, upper and lower limits of agreement, and percent error for vBMD.

### 3.3 Stiffness Derived From Internal Density Calibration

**Figure 5:**
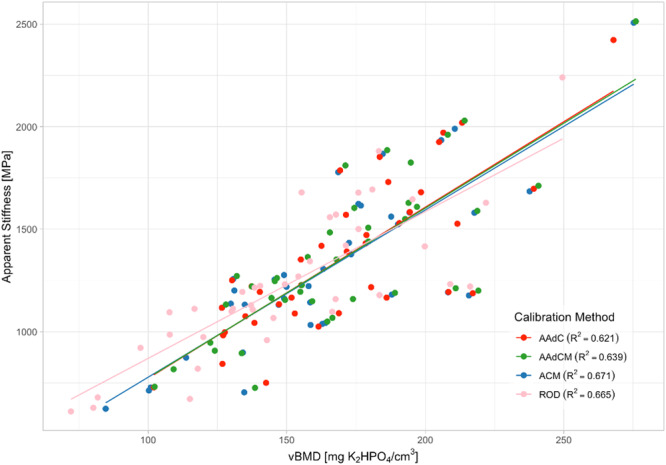
Correlations between vBMD [mgK₂HPO₄/cm³] and apparent stiffness [MPa] across calibration methods. Linear regression plots illustrate the relationships between apparent stiffness and vBMD derived from different internal calibration tissue combinations (AAdC, AAdCM, ACM), as well as from phantom calibration (ROD) (*n* = 39).

**Figure 6:**
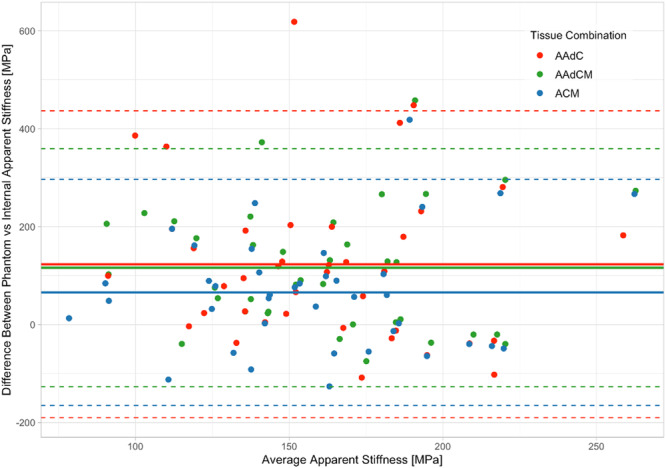
Comparing the stiffness derived from internal calibration techniques relative to phantom‐based calibration in the group of nonpathologic cadaveric images containing a phantom. Bland‐Altman plot for apparent stiffness [MPa] values for internal calibration according to tissue combination versus phantom calibration. Dashed lines indicate ± 1.96 standard deviations (SD) of the mean (solid line) (*n* = 39).

In the 39 nonpathologic cadaveric images containing a phantom, linear regression showed strong correlations between apparent stiffness and vBMD values for each calibration method (AAdC *R* ^2^ = 0.61; AAdCM *R* ^2^ = 0.63; ACM *R* ^2^ = 0.66; ROD *R* ^2^ = 0.66) and slopes not significantly different from 1 (AAdC: *y* = 8.34x–61.7; AAdCM: *y* = 8.28x–53; ACM: *y* = 8.15x–37.3; ROD: *y* = 7.16x + 154) (*p* < 0.001) (Figure 5). Bland–Altman analysis revealed the ACM tissue combination had the lowest error in estimated apparent modulus, compared to phantom‐vBMD derived FEMs, with a mean bias of 65.90 MPa (5.46%) and 95% limits of agreement ranging from −165.01 to 296.81 MPa (Figure 6, Table 3).

**Table 3:** Bland‐Altman results for each internal calibration tissue combination, relative to phantom calibration for apparent stiffness.

	AAdC	AAdCM	ACM
Mean difference (MPa)	123.30	116.39	65.90
Upper limit (MPa)	436.70	359.44	296.81
Lower limit (MPa)	−190.11	−126.66	−165.01
Percent error (%)	11.55	10.27	5.46

*Note:* The table shows mean differences, upper and lower limits of agreement, and percent error for apparent stiffness.

## 4 Discussion

This study evaluated whether internal density calibration methods could serve as a reliable alternative to phantom calibration for quantifying proximal humerus vBMD and estimating bone stiffness from single‐energy CT scans. Specifically, we compared vBMD values and FEM‐derived stiffness across three internal tissue combinations (AAdC, AAdCM, ACM) relative to phantom‐based calibration (ROD). Our findings demonstrate that internal calibration methods are strongly correlated with phantom‐derived vBMD, with the ACM combination (air, cortical bone, skeletal muscle) showing the highest agreement. ACM produced the lowest mean bias and narrowest limits of agreement in both vBMD and stiffness estimates, providing the highest agreement with the gold standard phantom calibration. Furthermore, all calibration methods preserved a relationship between vBMD and estimated stiffness, reinforcing the biomechanical relevance of internally calibrated images. These results support the use of internal calibration, particularly the ACM tissue combination, as a practical alternative in settings where phantom‐based calibration is unavailable.

Following the recommendation by Matheson et al. [24], each tissue combination used in this study included cortical bone and air. Research at the hip demonstrated that any combination using cortical bone and air as referent tissues yield comparable results to phantom calibration. The inclusion of air provides a stable and well‐established mass attenuation value that should be consistent between individual scans, while the use of cortical bone as a referent establishes an appropriate upper boundary for the linear regression framework underlying the internal calibration method [34]. Our results in the proximal humerus are consistent with findings in the hip, where the ideal tissue combination is air, skeletal muscle, and cortical bone [24]. Notably, the inclusion of skeletal muscle improved the agreement to phantom calibration, as demonstrated by the AAdC combination (air, adipose, cortical bone) having the lowest agreement. This is interesting given that the mean HU value for skeletal muscle in this dataset is lower than expected, with a large standard deviation. This discrepancy may explain the larger mean differences observed in our proximal humerus results compared to results in the hip [24]. One factor that could be contributing to the lower reported HU value in skeletal muscle may be the decreased hydration levels of the cadaveric specimens. Dehydration reduces water content, decreasing tissue density [43]. Since the musculature of the shoulder is generally smaller than that found in the hip, another contributing factor may be fat infiltration in the skeletal muscle ROIs, which would further reduce the HU value [44]. Given the lower mean and greater variability in skeletal muscle HU, further analysis is required to quantify muscle ROI placement on vBMD error, this will help guide future internal calibration protocol. Future studies should prioritize the selection of tissue ROIs that yield HU values within the expected range and exhibit minimal HU variation.

It is important to note that this internal calibration method was originally developed using patient images of living tissue, which may explain the higher mean difference in vBMD in the proximal humerus (ACM: 13.08 mgK_2_HPO_4_/cm^3^) relative to the hip (ACM: 1.13 mgK_2_HPO_4_/cm^3^). Future work should evaluate the performance of internal density calibration relative to phantom calibration in the proximal humerus of patients undergoing shoulder arthroplasty for end‐stage OA. If such studies yield results consistent with those observed at the hip, the internal density calibration method could provide a solution for estimating vBMD in patients undergoing shoulder arthroplasty for end‐stage OA, where phantoms are not present in the CT image. Additionally, while the use of images collected on a single scanner minimized confounding factors for this initial comparison between internal and phantom calibration methods, future work should incorporate different scanners and protocols to improve generalizability.

Results from the FEM further validate the use of the ACM internal calibration method over other tissue combinations in the proximal humerus when phantoms are not present in the image. However, vBMD only explained 66% of the variance in apparent stiffness for both phantom calibration and the ACM internal calibration method, highlighting the FEM's current limitations in fully capturing the complex mechanical properties of bone without experimental validation. This raises the possibility that FEM‐derived stiffness may not accurately reflect the true mechanical response of the proximal humerus to the stemless humeral component. While the density‐modulus relationship was derived from the literature [40], it has not yet been experimentally validated in the proximal humerus. Importantly, this relationship was defined using K_2_HPO_4_ density, minimizing potential confounding bias introduced through density conversion required by many other published density‐modulus relationships [40, 45]. While the CT image itself considers the density and composition across the specimen, the density‐modulus relationship is essential for establishing how the tissue will respond under stress. Further validation of the density‐modulus relationship might reveal the need to differentiate cortical and trabecular bone within the FEM. If cortical and trabecular bone exhibit different modulus values at a given density, a nonuniform, region‐specific density modulus relationship may be needed to accurately represent their mechanical behaviors.

Another limitation is that the FEM loading conditions unlikely replicate the loading of the stemless component on the resected plane. For model simplicity, this study does not account for shear or off‐axis forces, which may contribute to off‐axis loading due to the anisotropic nature of bone [46]. This simplification may bias stiffness relative to true implant–bone interactions by underestimating compliance and overestimating directional stiffness in anisotropic bone; future research should analyze directional sensitivity. Additionally, in the context of stemless shoulder arthroplasty, it may be important to consider the material properties of the stemless component itself and how it interacts with the bone. There are several studies which examine the different techniques for securing the implant with different backing designs to optimize load transfer, highlighting the importance of considering patient‐specific vBMD and morphology when selecting a suitable stemless implant [9, 47, 48].

This study provides initial site‐specific apparent stiffness values, which have yet to be established in the proximal humerus region. The novelty of this lies in applying the internal calibration method within the proximal humerus and utilizing these internally calibrated QCT images as FEM inputs.

## 5 Conclusion

The results of this study support the use of internal density‐calibrated images as valid inputs into FEMs for estimating stiffness in the proximal humerus. By linking vBMD with estimated stiffness values, this approach considers the mechanical properties of bone in the region supporting the humeral component, offering potential improvements to preoperative planning for stemless shoulder arthroplasty. Future research should validate this approach in vivo and refine the region‐specific density‐modulus relationships to improve FEM‐based preoperative planning in shoulder arthroplasty.

## Author Contributions

**Chloë K. A. Stiles:** conceptualization, methodology, software, investigation, formal analysis, data curation, writing – original draft preparation, visualization. **Bryn E. Matheson:** conceptualization, methodology, software, writing – review and editing. **George S. Athwal:** resources, writing – review and editing. **Jack P. Callaghan:** supervision. **Clark R. Dickerson:** supervision. **Steven K. Boyd:** resources, writing – reviewing and editing, supervision. **Nikolas K. Knowles:** conceptualization, writing – reviewing and editing, supervision.

## Ethics Statement

Ethics approval for retrospective review of cadaveric CT image data was reviewed and approved by the Western University WREM (#122657) & University of Waterloo REB (#45088).

## Conflicts of Interest

The authors declare no conflicts of interest.
